# Toxic Thyroid Adenoma Presenting as Apathetic Hyperthyroidism: A Case Report

**DOI:** 10.7759/cureus.61322

**Published:** 2024-05-29

**Authors:** Mina Hanna, Bo Sun, Ravindraprasad Shekarappa

**Affiliations:** 1 Medicine, Trinity School of Medicine, Warner Robins, USA; 2 Medicine, Trinity School of Medicine, Kingstown, VCT

**Keywords:** hyperfunctioning nodule, benign adenoma, apathetic hyperthyroidism, toxic thyroid adenoma, hypothalamic-pituitary-thyroid axis

## Abstract

The thyroid gland is an essential endocrine organ that secretes hormones to regulate homeostasis across multiple organ systems throughout the body. It is actively regulated by the hypothalamic-pituitary-thyroid (HPT) axis, where negative feedback modulates the amounts of active hormone being released; thus, lesions that disrupt the proper functioning of this gland or its regulatory mechanisms can be destructive. Toxic thyroid adenomas are usually singular benign functioning nodules in the thyroid gland that cause thyrotoxicosis. Hyperthyroidism is commonly clinically silent, however, in most symptomatic cases, patients will be diagnosed based on abnormal laboratory findings and typical hyperthyroid symptoms. This case report examines an 81-year-old male with an extensive medical history who presented with complaints of new-onset generalized fatigue coupled with bilateral lower extremity muscle cramps. A positron emission tomography (PET) scan for other medical conditions incidentally noted mildly increased uptake in the thyroid gland, prompting a further investigation that resulted in a diagnosis of toxic thyroid adenoma. The patient responded well to treatment with methimazole and has remained in a euthyroid state.

## Introduction

The thyroid gland, a vital endocrine organ located in the anterior neck posterior to the sternothyroid and sternohyoid muscles, plays a crucially active role in regulating and maintaining homeostasis in the body. It is typically found between the C5-T1 vertebral levels and encircles the cricoid cartilage and tracheal rings. The thyroid gland participates in the hypothalamic-pituitary-thyroid (HPT) axis via negative feedback mechanisms. The HPT axis, responsible for the production and regulation of thyroid hormones, functions to regulate respiration, cardiac rate/rhythm, euthermic state, metabolism, neuropsychiatric condition, and a plethora of other bodily operations. As a result of its imperative role in daily bodily functions, thyroid hormone production is a tightly regulated process that is controlled by multiple negative feedback mechanism loops [1].

Thyroid hormone production begins with the release of thyroid-releasing hormone (TRH) from the periventricular nucleus in the hypothalamus. In turn, TRH stimulates the adenohypophysis to secrete thyroid-stimulating-hormone (TSH), which subsequently prompts the thyroid gland to release thyroid hormones into the systemic circulation in the form of either 3,5,30,50-tetraiodothyronine (T4) and 3,5,30-triiodothyronine (T3) [1]. Notably, the majority of hormones released from the thyroid gland are in the inactive form of T4, with only 10% of hormones released being in the active form of T3 [2]. The inactive circulating thyroid hormone is subsequently converted into the active T3 form at target locations, thereby exerting effects in the periphery. As such, lesions and pathological processes of the thyroid gland can be detrimental if not diagnosed and treated promptly [3-5].

Lesions of the thyroid gland are certainly more prevalent than other pathologies, yet often present as clinically silent. Most patients remain asymptomatic with normal thyroid hormone output [3]; however, a certain subset of patients may present with symptoms consistent with either thyroid hormone overproduction or underproduction.

This report seeks to investigate the atypical symptomatic presentation of a toxic thyroid adenoma in an elderly male who presented with vague and non-specific symptoms, with the hopes of giving clinicians and students increased exposure to this pathology, aiding them to better identify and manage patients who may present similarly.

## Case presentation

An 81-year-old Caucasian male presented to the clinic with complaints of new-onset persistent generalized fatigue and bilateral lower extremity muscle cramps that were characterized as ongoing but mild in nature. The patient related that the symptoms had been worsening for two months prior to the visit with no aggravating or relieving factors. He noted that the muscle cramps were localized to his hip joints at a constant 5/10 intensity with no radiating pain. He reported no associated symptoms of heat intolerance, palpitations, diaphoresis, or unintentional weight changes; however, he stated that he had felt more tired than usual.

The patient had an extensive past medical history of chronic lymphocytic leukemia (CLL), small cell B-cell lymphoma, coronary artery disease (CAD) status-post coronary artery bypass grafting (CABG), hypercholesterolemia, essential hypertension, benign prostatic hyperplasia, gastroesophageal reflux disease, dysphagia, and prediabetes. His medication list included acalabrutinib 100 mg capsules twice daily, aspirin 325 mg daily, atorvastatin calcium 20 mg daily, lisinopril 10 mg daily, alfuzosin HCl 10 mg daily, and famotidine 20 mg twice daily. His surgical history included a cholecystectomy, CABG, colonoscopy, and cataract surgery. The patient denied the use of any alcohol, tobacco, or recreational drugs.

On physical examination, the patient appeared to be alert, well-nourished, and well-groomed with no acute distress. Neck palpation was unremarkable with a soft and non-tender thyroid with no palpable enlargements or abnormalities. Respiratory and cardiac examinations were within reference range and there was no suspicion of an arrhythmia or respiratory dysfunction. Musculoskeletal examination revealed normal muscle tone and strength bilaterally throughout all extremities with no visible gait abnormalities; deep tendon reflexes were normal.

Notably, the patient underwent a positron emission tomography (PET) scan prior to this visit as a follow-up on other comorbidities, which incidentally revealed a mildly increased uptake in the right lower lobe of the thyroid gland. Routine blood work conducted during a recent annual physical examination revealed suppressed levels of TSH coupled with an increase in free T4, thyroid peroxidase (TPO) antibodies, and thyroglobulin antibodies (Table 1). These abnormal findings prompted further investigation with an iodine-123 uptake scan.

**Table 1:** Results from a thyroid laboratory panel conducted during routine bloodwork

	Patient X's pre-treatment values	Normal range
TSH	<0.03 uIU/mL	0.340 to 5.600 uIU/mL
Free T4	1.65 ng/dL	0.610 - 1.120 ng/dL
TPO antibodies	11 IU/mL	< 9 IU/mL
Thyroglobulin antibodies	235 IU/mL	< or = 1 IU/mL

An iodine-123 (I-123) uptake scan was subsequently performed with 127.5 uCi of I-123 via the oral route. Results showed an increased uptake of radioactive iodine in the thyroid gland after two hours and six hours post-infusion when compared to normal reference uptake values (Table 2). These findings pointed to the inclusion of Graves disease and toxic thyroid adenoma as part of the differentials.

**Table 2:** Findings from a radioactive iodine uptake study using iodine-123

	Patient X	Normal Range
2-Hour Value	9.7%	3-7%
6-Hour Value	22.2%	5-15%

**Figure 1:**
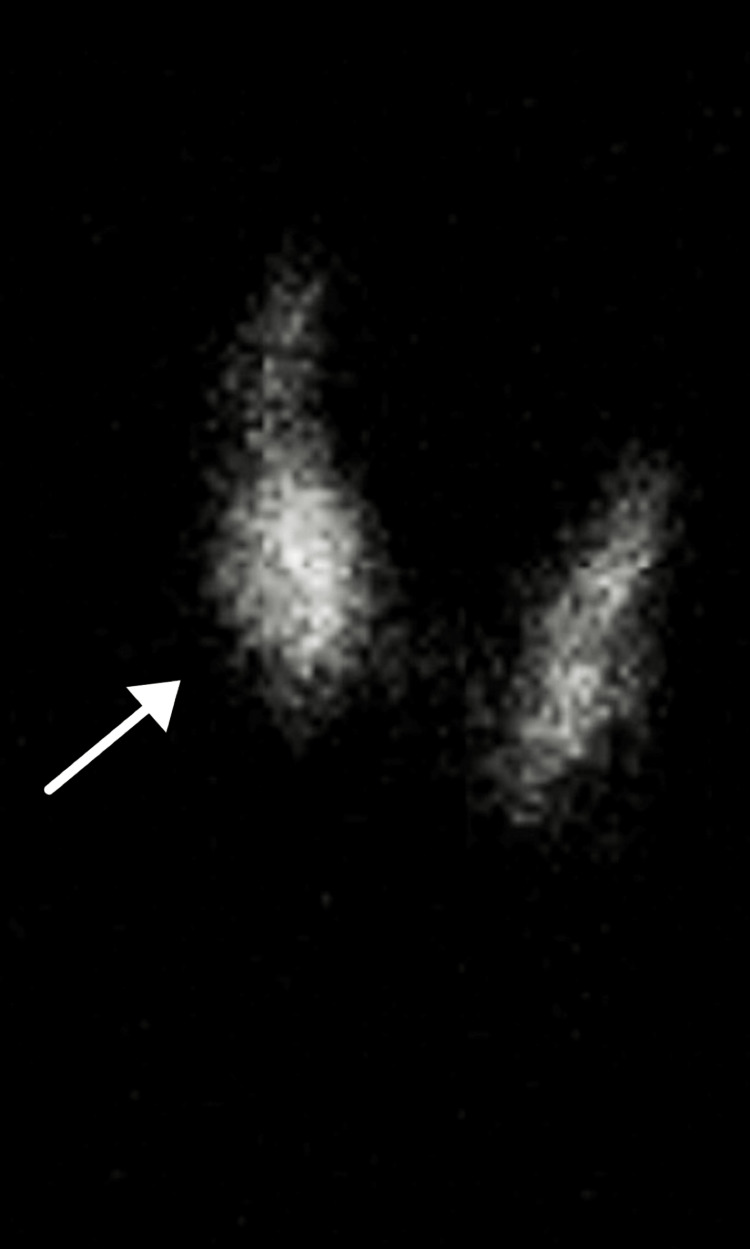
Radioactive iodine-123 uptake study results of the right anterior oblique (RAO) view of the thyroid gland *This image was obtained with the expressed verbal consent of the patient and the affiliated hospital.

An increased uptake was documented in the lower pole of the right lobe of the thyroid (Figure 1), and these findings were consistent with the diagnosis of a toxic thyroid adenoma. The patient was subsequently treated with methimazole 5 mg orally twice a day. This dose proved to be effective in this case and returned the patient to a euthyroid state.

## Discussion

This case report explores the atypical presentation of an 81-year-old Caucasian gentleman who presented with chronic ongoing generalized fatigue and bilateral lower muscle weakness. Due to abnormal routine blood work coupled with previous incidental findings of a mildly increased uptake in the right lower lobe of the thyroid gland on a positron emission tomography (PET) scan, further work-up with an I-123 uptake scan was warranted and confirmed the diagnosis of a toxic thyroid adenoma in the right lower lobe of the thyroid gland. The patient was subsequently treated with methimazole and responded well to the medication.

This case report seeks to investigate the varying presentations of toxic thyroid adenomas, particularly the atypical features reported in this elderly male.

Presentation and evaluation

The majority of toxic thyroid adenomas are usually clinically silent, which results in patients often being unaware of having this lesion. In most cases, patients will even be clinically euthyroid and rarely present with noticeable or palpable lesions in their neck. Even more scarcely will patients experience symptoms consistent with an abnormal thyroid state [6]. Typically, a complete and detailed physical examination along with a thyroid laboratory workup will reveal abnormalities, prompting further evaluation and exploration. In the presence of symptoms, patients typically complain of dyspnea secondary to compression of the trachea by a neoplastic lesion or thyroid tissue overgrowth, hoarseness of the voice due to irritation of the recurrent laryngeal nerve, or dysphagia secondary to esophageal compression [7]. Approximately 1% of patients will present with typical hyperthyroid symptoms such as unintentional weight loss, anxiety, cardiac palpitations, diarrhea, hair loss, and menstrual irregularities in pre-menopausal women [2,3]. This patient only reported symptoms of generalized fatigue and diffuse muscle weakness. The lack of typical hyperthyroid symptoms and the presence of these generalized complaints in an elderly patient would not prompt a clinician to suspect a toxic thyroid adenoma.

Patients who are suspected to have thyroid pathology will undergo a diagnostic workup that begins with an evaluation of TSH levels to assess the status of the thyroid gland. Patients with a toxic thyroid adenoma will have decreased levels of TSH along with elevated levels of thyroxine [7]. Further evaluation will include ultrasound imaging of the thyroid gland and a fine-needle aspiration (FNA) biopsy when warranted. In the case of this patient, a PET scan was conducted as part of the management of other comorbidities. The incidental finding of a mildly increased uptake in the right lobe of the thyroid gland prompted further evaluation of TSH and thyroxine levels. The abnormalities found in these laboratory values (Table 1) necessitated further diagnostic testing with an iodine-123 uptake scan, which subsequently revealed the presence of a hyperfunctioning nodule. 

It is important to note that hyperfunctioning nodules are considered to be benign with a very low probability of malignancy while non-functioning nodules have an increased risk of malignancy with approximately 20% becoming malignant [7]. For this reason, patients with nodules greater than 1 cm in size, a strong family history of differentiated thyroid cancer, young patients, or a history of radiation therapy in the head or neck are prime candidates for FNA biopsies [8,9]. This elderly patient did not meet any of the criteria stated by the American Thyroid Association; therefore, an FNA biopsy was not indicated.

Fine-needle aspirations of the majority of thyroid adenomas show follicular epithelial cells arranged in an organized manner; however, some may show abnormal architecture in the cellular arrangement. A toxic thyroid adenoma is only definitively diagnosed by biopsy when it is determined that there is no capsular or vessel invasion on histological examination following a thyroidectomy [7,8]. Because of this patient’s age, extensive medical history and comorbidities, and asymptomatic presentation, a thyroidectomy was not indicated in this case.

Incidence and prevalence

In the United States, the prevalence of hyperthyroidism is 0.035% in males and 0.102% in females. Notably, this prevalence rate increases to 0.058% in men above the age of 80; however, it decreases to 0.0718% in females above the age of 80. The overall prevalence of toxic adenomas is between 5-7%, accounting for approximately 1-2% of all hyperthyroid cases in men and women [10]. It is well known that the risk of pathological processes increases with age, and the thyroid is certainly not spared. Incidences of toxic adenomas have been found to increase with age, with the median age of diagnosis for men being above the age of 50. Of note, women are 5.9 times as likely to develop a toxic adenoma when compared to men, similar to trends seen in the development of autoimmune-related disorders [10,11].

It is well understood that iodine plays an essential role in thyroid hormone production and that being in an iodine-deficient state has profound impacts on thyroid function. For this reason, toxic thyroid adenomas are more commonly seen in countries with a higher prevalence of iodine deficiency. The prevalence of iodine deficiency is relatively low in the United States due to the implementation of iodized salt in 1942 [12].

Pathogenesis and histology

Hormonal production and suppression are predominantly regulated by negative feedback mechanisms that effectively control the levels of thyroid hormone in circulation. TSH, T3, and T4 participate in the HPT axis, which tightly regulates the thyroid gland and its hormonal production. In fact, TSH activates two different pathways to regulate T3 and T4 production in the thyroid gland. Upon binding to its receptor on the surface of thyroid follicular cells, TSH stimulates the conversion of adenosine triphosphate (ATP) to cyclic adenosine monophosphate (cAMP) via the heterotrimeric guanine nucleotide-binding protein alpha-subunit (Gs-alpha subunit protein). This enzymatic cascade leads to thyroid cellular growth and thyroid hormone secretion [13].

An increase in circulating T3 and T4 levels results in a negative feedback response on TSH secretion from the adenohypophysis. However, decreased T3 and T4 levels induce a drastic increase in TSH secretion. In the presence of an elevated TSH level of between three to five times the normal amount, there will be an activation of a G-q protein that prompts the enzymatic activation of phospholipase C, and, with the use of intracellular calcium, cleaves phosphatidylinositol 4,5-bisphosphate (PIP2) to form inositol phosphate (IP3) and diacylglycerol (DAG). This pathway regulates the iodination of thyroid hormone during its production [13].

A proposed mechanism for the formation of toxic thyroid adenomas is based on the malfunction of the G-s protein pathway that was described. A gain-of-function mutation in the germline or somatic cells of the thyroid gland results in a constitutively active G-s protein, thereby resulting in an overproduction of thyroid hormone that is unregulated and unresponsive to differing levels of TSH. This, in turn, also causes growth of the thyroid gland itself, resulting in palpable nodules or goiters that are clinically observable [11,14].

Treatment and management

The treatment and management of a toxic thyroid adenoma are typically achieved by either pharmacological suppression of the thyroid gland or surgical excision of the organ itself. Indication for treatment is subdivided into three categories: 1. Symptomatic hyperthyroidism: low TSH levels with elevated T3/T4; 2. Subclinical hyperthyroidism: low TSH levels with normal T3/T4; 3. Large goiters with symptoms of obstruction: may or may not have normal thyroid levels.

The treatment of symptomatic and subclinical hyperthyroidism begins with the management of symptoms that are distressing to the patient. In such cases of overt hyperthyroidism affecting the autonomic nervous system, treatment with selective and non-selective beta-blockers is initiated, in the absence of contraindications, prior to any diagnostic labs, procedures, or scans [15]. Pharmacological modalities of treating hyperthyroidism involve the use of medications such as thionamides: methimazole and propylthiouracil. These medications function to decrease thyroid hormone production; however, they do not cause disease remission [16,17]. Ideally, patients would undergo surgical or radioiodine ablation therapy for permanent treatment. Patients who are not ideal candidates for these treatment options would thus benefit from medical treatment instead [16].

The American Thyroid Association has published guidelines regarding the treatment of hyperthyroidism, reflecting the importance of medical treatment being a collaboration between practitioner and patient; often influenced by several factors unique to each case such as patient preference and expert availability. Patients with large goiters, symptomatic compression or obstruction of anatomical structures, or an emergent need to return to a euthyroid state, all make for good surgical candidates [18]. Other influences of treatment modalities include a patient’s concern for radioiodine treatment, general anesthesia, or surgical complications. In the absence of these factors, patients are better served to undergo a total or partial thyroidectomy to permanently remove the lesion. However, if a patient is not a good surgical candidate and has no emergent risk factors, prolonged thionamide therapy is acceptable with patient compliance and tolerance [17].

This patient’s age and multiple comorbidities, such as CAD status post CABG and disseminated cancer, make him an unlikely candidate for surgery. A revised cardiac risk index (RCRI) for this patient indicates that he is Class II risk, with a 6% likelihood of a cardiovascular event within 30 days should he undergo thyroid surgery. As a result, ongoing medical therapy was initiated with methimazole and has proven to be effective in managing his symptoms with good compliance and tolerance.

Further investigations

While toxic thyroid adenomas have been extensively studied to date, the pathogenesis of this condition is still in question. A proposed mechanism for the development of toxic thyroid adenomas involves a constitutively active G-s protein, resulting in the overproduction of thyroid hormone. However, there have been other potential associations, notably with low-salt diets, such as the DASH (dietary approaches to stop hypertension) diet, other concomitant autoimmune disorders, or other neoplastic lesions, thus warranting further investigation.

In this particular case, this patient has a prolonged history of CAD status post CABG, essential hypertension, and other comorbidities. It is highly likely, given his history, that the patient was following a low-sodium diet (DASH diet) as part of his treatment regimen. Iodine deficiency has been known to be the leading cause of thyroid abnormalities, and it is for this reason that the implementation of iodized table salt was initiated in 1942 in the United States; this has significantly decreased the prevalence of goiter. Patients placed on low-salt diets are potentially susceptible to inadequate iodine intake via dietary methods, thus placing them at increased risk for the development of thyroid pathologies [12].

Another potential link associated with the development of a toxic thyroid adenoma is female gender. Medical evidence backed by population statistics has shown that women are 5.9 times more likely to develop toxic thyroid adenomas than men [10]. This has been likened to how women are more likely to develop autoimmune disorders in comparison to men. This proposes the question of the impact of gender in developing certain conditions, particularly toxic thyroid adenomas. It also begs the question as to why an elderly male would develop this adenoma suddenly, not following the common trend.

Finally, another unique characteristic of this case was the nature of how this pathology was discovered in this patient. This patient was being managed for small cell B-cell lymphoma and had undergone a PET scan for that condition, which incidentally showed a toxic thyroid adenoma. Small cell B-cell lymphoma is the proliferation of lymphoid cells, B-cells in this case, to form a neoplastic lesion. This prompts the question of whether there is an added risk of developing other non-related neoplastic pathologies in the presence of a diagnosed neoplastic lesion. It is known that non-Hodgkin’s lymphoma spreads via the lymphatic system, and it is therefore worth investigating if this modality of spread has any impact on the subsequent development of cancerous growths in other body organs, including the development of toxic nodules. While these potential associations have not been medically validated, it is interesting to note these findings and the possibilities of their linkage.

## Conclusions

This case report examines an elderly gentleman with an incidental finding of a toxic thyroid adenoma with atypical features. The goal of this report is to present an atypical presentation of hyperthyroidism with the hopes of increasing exposure to students and clinicians, to be able to more effectively recognize it prior to symptomatic progression.

Hyperthyroidism is typically clinically silent and usually only diagnosed incidentally with annual laboratory tests. However, in the setting of symptomatic presentation, further investigation is warranted to identify the underlying pathology. The thyroid hormone normally functions to maintain proper respiration, cardiac rate/rhythm, euthermic state, metabolism, and neuropsychiatric status. As such, pathologies involving this hormone can be potentially devastating. Hyperthyroidism symptoms can vary between patients, but classic presentations include heat intolerance, unexplained weight loss, agitation, cardiac palpitations or arrhythmias, and much more. This patient's presentation consisted of atypical features, in that he presented with vague and generic symptoms of generalized fatigue and diffuse muscle cramping. These symptoms can be attributed to many of his other comorbidities or as a result of the normal aging process. He was only diagnosed with a toxic thyroid adenoma incidentally after undergoing a PET scan for small cell B-cell lymphoma follow-up; thus, highlighting the importance of recognizing these vague symptoms as possible effects of hyperthyroidism. Thyroid hormone production is a tightly regulated process, highly controlled by the hypothalamic-pituitary-thyroid axis. While this case report examines atypical features of this benign pathology, it opens the door for future investigations to determine the underlying causes of developing a toxic thyroid adenoma.
